# Ontological security and connectivity provided by pets: a study in the self-management of the everyday lives of people diagnosed with a long-term mental health condition

**DOI:** 10.1186/s12888-016-1111-3

**Published:** 2016-12-09

**Authors:** Helen Brooks, Kelly Rushton, Sandra Walker, Karina Lovell, Anne Rogers

**Affiliations:** 1 Division of Nursing, Midwifery and Social Work, Faculty of Biology, Medicine and Health, University of Manchester, Jean McFarlane Building, Oxford Road, Manchester, M13 9PL UK; 2 NIHR CLAHRC Wessex, Faculty of Health Sciences, University of Southampton, Southampton, UK

**Keywords:** Mental health, Social network mapping, Pets, Qualitative research, United Kingdom

## Abstract

**Background:**

Despite evidence that connecting people to relevant wellbeing-related resources brings therapeutic benefit, there is limited understanding, in the context of mental health recovery, of the potential value and contribution of pet ownership to personal support networks for self-management. This study aimed to explore the role of pets in the support and management activities in the personal networks of people with long-term mental health problems.

**Methods:**

Semi-structured interviews centred on ‘ego’ network mapping were conducted in two locations (in the North West and in the South of England) with 54 participants with a diagnosis of a long-term mental health problem. Interviews explored the day-to-day experience of living with a mental illness, informed by the notion of illness work undertaken by social network members within personal networks. Narratives were elicited that explored the relationship, value, utility and meaning of pets in the context of the provision of social support and management provided by other network members. Interviews were recorded, then transcribed verbatim before being analysed using a framework analysis.

**Results:**

The majority of pets were placed in the central, most valued circle of support within the network diagrams. Pets were implicated in relational work through the provision of secure and intimate relationships not available elsewhere. Pets constituted a valuable source of illness work in managing feelings through distraction from symptoms and upsetting experiences, and provided a form of encouragement for activity. Pets were of enhanced salience where relationships with other network members were limited or difficult. Despite these benefits, pets were unanimously neither considered nor incorporated into individual mental health care plans.

**Conclusions:**

Drawing on a conceptual framework built on Corbin and Strauss’s notion of illness ‘work’ and notions of a personal workforce of support undertaken within whole networks of individuals, this study contributes to our understanding of the role of pets in the daily management of long-term mental health problems. Pets should be considered a main rather than a marginal source of support in the management of long-term mental health problems, and this has implications for the planning and delivery of mental health services.

## Background

The ability to manage and to be engaged with everyday life is a key concern of people with a long-term condition, which also applies to people experiencing mental health problems. Losing previous connectivity and perceived social status with people, losing valued activities and experiencing feelings of loneliness and isolation have been well documented as ongoing concerns [1, 2]. Related to these concerns is a sense of ontological security, which refers to a sense of order and continuity derived from a person’s capacity to give meaning to their lives and to maintain a positive view of the self, world and future [3]. The latter is considered to require positive and stable emotions and the avoidance of chaos and anxiety [3]. In relation to mental health, ontological security is threatened by the breakdown of, and difficulties in, maintaining relationships with friends and family [4], challenges in maintaining routine and daily living activities [5], and feelings of being judged and stigmatised [6, 7].

Having a support network in place provides options for the management of living everyday life with a mental health problem. In this respect, emphasis is often placed on family, friends, and social interaction with other people [8–10]. However, the role of pets is likely to have been under-acknowledged, with indications in research that some people consider their pets as being as important as family members, and their value in terms of companionship, love and support is widely acknowledged [11]. Analysis of an individual’s support network suggests a unique contribution from pets that extends beyond the support and connections provided by familial, friendship and weak tie connections. Weak ties are characterised by relatively brief interactions with acquaintances and strangers but represent important sources of support and are attributed with the power to enhance the reach and cohesion of other social relations [1, 10].

Confirmatory evidence of the multifaceted relationships that exist between people with health problems and their pets emanates from the analysis of narrative accounts which illuminate the presence of, or talk about pets, as producing differing reactions from those of other household members [12]. There has also been recognition of the more distal benefits that accrue from pet ownership, including the benefits to and from the broader community and through the building and receipt of social capital [13]. Social capital refers to the social, economic and cultural resources on which individuals draw in responding to long-term health conditions. These represent resources that form an integral part of people’s social networks, which are impacted upon by wider determinants of health [14]. Class-related cultural resources interact with economic and social capital in the structuring of people's health chances, choices, and the unequal distribution of health outcomes [15].

In terms of mental health, the value of the broader role of animals is demonstrated in Animal Assisted Therapy (AAT), which has been found to be effective in psychiatric inpatient populations [16] and residential care settings [17]. However, despite AAT gaining popularity in recent years, and therapy animals becoming increasingly familiar sights in care homes, hospices and hospital wards, pets are not considered in care planning processes undertaken for managing mental health on an on-going basis. This may in part be due to a gap in evidence or in evidence failing to inform or reach practitioners and policy makers responsible for care planning arrangements. Whilst the benefits of formalised AAT for conditions such as dementia [18, 19], cancer [20, 21] and childhood developmental disorders [22, 23] are gaining recognition, there is currently a lack of evidence exploring the contribution of pets in the broader context of support networks and the role they may play in recovery-orientated activities and the management of mental health.

Studies have examined the benefits of owning and caring for pets demonstrating reduction in stress [24], improved quality of life [25, 26], improved physical health [27–29], increased social interaction [30] and reduced loneliness [2, 31].

The current study aimed to develop an understanding of the meaning and roles credited to pet ownership and engagement by those with a diagnosis of mental illness within the wider context of recovery activities and the role of other members of individuals’ personal communities. Previous research has demonstrated the utility of pets for mediating social connections linked to the mobilisation of resources for those with long-term physical conditions [32]. Here we extend the focus of this previous analysis to the role of pets for mental illness, which is currently equivocal and underexplored.

## Methods

This paper reports on the findings from qualitative interviews focussed on ‘ego’ network mapping to elicit an understanding of personal support derived from social network members conducted in two locations; Manchester and Southampton. The nature of support provided by social network members and the wider community in the management and everyday experience of living with a mental illness was explored.

The methods have been informed by the consolidated criteria for reporting qualitative studies (COREQ) guidelines [33]. The design and analysis of the study used a conceptual framework which built on Corbin and Strauss’s notion of illness work [34] and notions of a personal workforce of support undertaken within whole networks of individuals with chronic illness (Table 1). This approach allows for a close inspection of what tasks are undertaken to manage illness, who does them, how and where these activities are undertaken and also identifies any potential problems associated with this ‘work’ [34].

**Table 1:** The illness work framework

Types of work			Definitions
Practical work	Practical Illness work		Work related to health management.
		Contingency/improvisation	Crisis prevention and management: ‘work that gets things back “on track” in the face of the unexpected, and modifies action to accommodate unanticipated contingencies’ (potential support).
		Translation, mediation and embodiment	The translation of abstract knowledge into practical knowledge and then into practice. The difference between knowing and doing. Includes illness-specific work related to diet, exercise and medication (regimen work). Symptom management and diagnostic-related work related to assessment of health status.
		Coordination work	Involves combining different entities such as tasks, types of work and people, making them work together within a specific context. Also involves negotiations regarding the ways in which work is done, who does what, when, how and why. The organisation of tasks that need to be done.
		Advocacy work	The negotiation of contributions and the work done by others on one’s behalf.
	Practical everyday work		Housekeeping and repairing; occupational work; child rearing; sentimental work; eating. Includes generic support related to diet and exercise (general shopping and unspecific personal care).
		Everyday work–diet	Work related to non-specific, diet-related support (shopping, cooking, going for a meal).
		Everyday work–exercise	Work related to non-specific, exercise-related support (walking, swimming, going to the gym).
Emotional work	Illness specific emotional work		Work related to comforting when worried or anxious about health-related issues.
	Everyday emotional work		Work related to comforting when worried or anxious about everyday issues. Well-being and companionship.
Biographical work	Biographical work		Work related to the actions taken to retain control over the life course and to give life meaning again. This includes the reassessment of personal expectations, capabilities, future plans, identity, relationships and strong emotional bonds. Includes illness-related and non-illness related biographical events.

Drawn from the work of Corbin and Strauss [31]

The definitions of the categories of work included in this study can be found in Table 2 and were combined as follows: practical, emotional and biographical work. The notion of illness work was preferred to alternative theories of social support as it provides a useful lens through which to understand the resources, networks and relationships associated with the management of severe mental illness and allows participants to self-identify a wide range of contributors relevant to their unique circumstances [1, 10, 32].

**Table 2:** Definitions of types of work used within the paper

Practical work	Work related to housekeeping and repairing; occupational work; child rearing; support and activities related to diet and exercise, general shopping and unspecific personal care. In addition, practical work incorporates the work related to taking medications, crisis prevention and management, regimen work, taking and interpreting measurements, understanding symptoms, making appointments, etc.
Emotional work	Work related to comforting when worried or anxious about everyday matters, including health, well-being and companionship.
Biographical work	Work related to the actions taken to retain control over the life course and to give life meaning again. This includes the reassessment of personal expectations, capabilities and future plans, personal identity, relationships and biographical events.

Drawn from the work of Corbin and Strauss [31]

### Recruitment

Participants were recruited from 1) a randomised controlled trial exploring service user and carer involvement in mental health care planning (EQUIP, Manchester) and 2) a sample of people using a Recovery College (Southampton). Participants were recruited via invitation letters and flyers advertising the study. Those who were interested in taking part contacted the research team directly to discuss the study in more depth and then arranged a convenient time, date and location for interview. Informed, written consent was obtained prior to the interview. Purposive sampling was used to select participants to allow for diversity in terms of age and gender. Recruitment stopped upon agreement amongst the study team that theme saturation had occurred and there was consensus that no new themes were arising from the data.

### The sample

Participants were considered eligible for inclusion in the study if they were aged 18 or above, were under the care of community-based mental health services (or had been discharged within 6 months) and had received a diagnosis from a health professional of a severe mental illness (e.g. Schizophrenia or Bipolar disorder).

Twenty-nine participants were recruited to the study in Manchester (12 of whom identified a pet in their social network) and 25 participants were recruited from Southampton (13 of whom identified a pet in their network). See Table 3 for more detail on study participants.

**Table 3:** Participant characteristics (those with pets *n* = 25)

Characteristics	Number	Percent
Gender		
Female	17	68 %
Male	8	32 %
Location		
Manchester	12	48 %
Southampton	13	52 %
Ethnicity		
White	25	100 %
Non-white	0	0 %
Number of pets		
1	16	64 %
2	5	20 %
3	0	0 %
4	3	12 %
5+	1	4 %
Type of pets		
Dog only	7	28 %
Cat only	8	32 %
Bird only	2	8 %
Hamster only	1	4 %
Guinea pig only	1	4 %
Mixture	4	16 %
Not specified	2	8 %

### Data collection

Face-to-face, semi-structured network interviews were carried out between March 2015 and February 2016 by either HB or SW at participants’ homes or an agreed local community facility. Participants were asked to map personal networks using a diagram, which consisted of three concentric circles [35]. Interviewers started the interview by asking the question *‘Who or what do you think is most important to you in managing your mental health?*’. Participants could place nominated network members in either the central circle considered *most important*, the middle circle, considered *important but not as important as the central circle* or the outer circle, considered *important but not as important as the two more central circles*. Identified network members included friends, family members, health professionals, pets, hobbies, places, activities and objects. There was no maximum number of network members imposed on participants and they were free to list as few or as many as they considered relevant to their unique situation.

The interviews lasted between 20 and 90 min and explored the role and key attributes of individual network members to mental health management based on the aforementioned categories of work (see Appendix 1 for an interview schedule). This way, detailed information was collected about the contributions each network member made to the different types of work associated with mental health management. Interviews were digitally audio-recorded, transcribed verbatim, anonymised and allocated to a member of the study team (HB, KR, SW alternatively) for analysis.

HB and KR are health service researchers, SW is a Lecturer in Mental Health, KL is a Professor in Mental Health and AR is a Professor of Health Systems Implementation. As such, researchers had no therapeutic relationship with participants. The conceptual starting point of our study is one informed by a capabilities approach which recognises that the social context and engagement with valued people, places and activities are often hidden from view but are likely to be as important to the management of long-term conditions as traditional therapeutic or self-management support approaches [36].

### Data analysis

Transcripts were read a number of times to ensure familiarisation. Excel software was used to aid analysis along with a paper trail detailing framework development contained in a word document for transparency purposes.

A framework analysis was undertaken with individual members of the study team coding data relating to work-related codes (practical, emotional and biographical work, see Tables 1 and 2 [34]) implicated in narratives about the role of pets. Each author (HB, KR and SW) coded transcripts independently and a subset of transcripts were independently analysed by AR, with any coding discrepancies discussed amongst the team to enhance rigour and trustworthiness of data. Researchers met regularly to discuss on-going analysis and to discuss, explore and confirm emergent codes and to remove duplicated codes.

Network diagrams were analysed descriptively to identify the size of network, whether a pet was in the network, along with the relative position of the pet within the network. The study took an individual network approach to understand how the participant managed their condition and the types of support they utilised across the network including the comparative contribution of pets. The main themes that emerged from the coding were the placement of pets and associated attributional meaning within personal communities; the nature and balance of emotional, illness and biographical work; and the hidden work of pets.

## Results

### Network placement and attributional meaning of pets

**Fig. 1:**
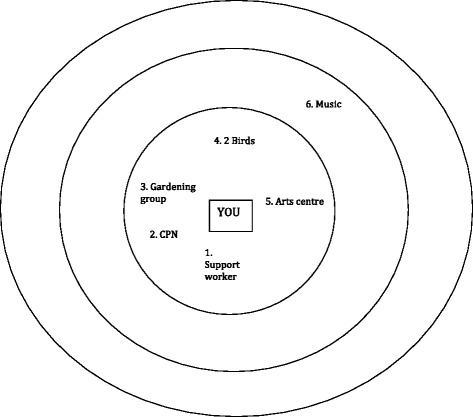
Example Network Diagram (ID 2)

Of the 25 participants who identified a pet within the personal communities associated with the management of mental health and everyday life, the majority (60 %, *n* = 15) placed their pet in the central most important circle. A further 20 % (*n* = 5) placed their pet in the second circle and 12 % (*n* = 3) placed their pet in the third circle. The remaining 8 % (*n* = 2) whilst identifying a pet within their social network did not place them in one of the three concentric circles. Figure 1 details the network diagram completed by ID 2. This male participant had a relatively small network (*n* = 6) in which his pet birds were placed in the central, most important circle. The only human members of his network were his Community Psychiatric Nurse (CPN) and support worker whom he saw infrequently, highlighting the importance of his pets for the management of his mental health.

Examples of animals cited as relevant to the management of mental health included family pets, working animals and more peripheral links to animals in the wider community in places such as urban farms and animal rescue centres. The prominence and salience of animals within an individual’s personal community network varied. Some individuals had networks dominated by pets, coined ‘pet centric’ networks, which provided a range of direct and indirect benefits, whilst others had one animal positioned in a fairly peripheral position within the network (Appendix 2). It was often the case that where relationships with family and friends were seen to be good, animal-human relationships were perceived to be of secondary importance. However, the majority of people reported either having difficult relationships with other network members including friends and family or had little or limited other network support in addition to their pets. For these people, the relationships with companion animals took on discrete and definite functions within networks, which were different to the norms associated with human-to-human relationships. These appeared to centre on the receipt of ontological security not available from elsewhere, as well as physical proximity and consistency when compared to the other relationships.

*So with my pets I suppose although my Mum and Dad are very significant figures they’ve also got their own lives and lots of other things going on so I’m only one aspect of that life and I feel that the pets I suppose they depend on me and also I have daily contact with them and they also give me a sense of wellbeing which I don’t get from any [one else] because most of these interactions with my Mum, Dad, [friend], are all by telephone rather than physical contact and that’s the big difference is the empathetic physical presence. *(ID 21, 10 birds, first circle)

#### Relational work and substitution

Relational work has been used to describe the tasks that are required to develop and sustain interpersonal relationships [37]. A core theme that arose from the data during narratives was the attributional meaning of relationships with pets. Some invested energy in a singular focus on a preferred pet. For those without close friends and family, the intensive and positive identification associated with their pets made for intense, intimate relationships ‘the relationship with my cat was the only thing that stayed constant’ (ID 7, one cat, second circle). Individuals often saw their pet most frequently and for some, they were their only source of support. In this way, pets featured highly in the network hierarchy and were linked to dependency and substitutability of other, often absent network members providing or replacing ontological security from other sources.

*Well I just love animals, I just really do love animals. I haven’t got a partner so I have something around me otherwise I’d go totally bonkers. That’s the most important thing to me is my animals* (ID 13, range of pets, first circle).

Participants described the various, nuanced ways that pets connected them to others in, and beyond, their personal networks or to the wider social environment. Participants described new relationships with network members or community organisations as a result of pet ownership, as well as enhanced ones with existing network members.

*That surprised me, you know, the amount of people that stop and talk to him, and that, yeah, it cheers me up with him. I haven’t got much in my life, but he’s quite good, yeah* (ID 9, 1 dog, first circle).

For participants in this study, the connection an individual felt with their pet was seemingly of relatively more salience when compared to studies of other long-term conditions [32]. Linked to ontological security, participants spoke in depth about the connection they felt with their pet, which was often not replicated in their relationships with friends and family, either because they had no human network members or existing relationships were difficult.

*If I didn’t have my pets I think I would be on my own…You know what I mean, so it’s…it’s nice to come home and, you know, listen to the birds singing and that, you know* (ID 2, 2 birds, first circle).

*I felt in a sense that my cat was my familiar in that he understood or was an extension of my thoughts *(ID 7, 1 cat, second circle).

These limited or difficult connections with others were often perceived to have come about from what participants referred to as a ‘gulf in understanding’ between themselves and the other humans in their network. Participants felt that in order to have a beneficial relationship with friends and family, there needed to be a shared understanding of their mental health condition, which was problematic to obtain without direct experience and in the absence of similar value judgements or thwarted expectations. Pets, on the other hand were credited with either having an understanding of their owners’ mental health problems without the need for this to be communicated, or as being a network member with whom they could have an adequate relationship without this pre-requisite level of understanding.

*I think it's hard really when you haven't had mental illness to know what the actual experience is for someone who has had the experience. There's like a chasm, deep chasm between us - a growing canyon. They're on one side of it and we're on the other side of it. We're sending smoke signals to each other to try and understand each other but we don't always - we don't always understand each other I don't think. *(ID 1, 1 cat, first circle).

One important component of the relationship with pets was a sense of enduring trust between individuals and their companion animal, which enhanced the value of pets when compared with humans. Often, participants described fractured relationships with friends and family that had occurred due to past behaviour on their part attributed to mental illness, which had caused existing relationships to strain or to break down. Participants also worried about upsetting the humans in their networks. It was considered that pets, on the other hand, were not subject to these sensitivities and thus possessed the capacity to form more enduring and secure relationships. People talked about pets still being there however they were treated and compared this directly to relationships with friends and family. In this way, pets served to provide a unique form of validation through unconditional support, which was often not forthcoming from other network relationships.

*Er, there’s a lot less things to worry about. I mean you can’t…you can’t like be like if he was naughty or anything like that you’d tell him off and that was it and there’d be no hard feelings. That there’s not, you don’t get the nastiness* (ID 11, 1 dog, third circle).

Alternatively, participants provided examples of friends and family not having been a helpful or useful source of support in difficult times, which meant they were reluctant to trust or to rely on them to provide this in the future. Additionally, participants alluded to a general distrust of people attributable to a sense of vulnerability attached to mental illness. These concerns became heightened during times of acute mental health crises.

*Yes, they can give you loving, pets can and you can trust pets not to steal off you* (ID 6, 1 guinea pig, first circle).

Frequently, participants expressed the view of wanting to avoid the world when acutely unwell, whilst at the same time acknowledging that this was sometimes a direct barrier to recovery. Pets provided participants with a mechanism for engagement with the social world through having to care for their animals no matter how they felt. This sense of purpose was considered fundamental to a sense of wellbeing and recovery and demarcated the support provided by pets from that given by other network members, which was often considered conditional on moment-to-moment changes in a person’s mental health status (e.g. only seeing friends when feeling well enough).

*You know, so in terms of mental health, when you just want to sink into a pit and just sort of retreat from the entire world, they force me, the cats force me to sort of still be involved with the world* (ID 5, 2 cats, first circle).

### Balancing of emotional, illness and biographical pet work

#### Negative work and burden

In a small number of instances, negative aspects to pet ownership surfaced. These ‘deviant’ cases included narratives related to the burden of looking after pets, pets as a source of anxiety and the acknowledged or anticipated distress when loved companion animals died. Additionally, whilst pets were identified as a valuable source of support in times of crisis, one participant talked about her pets blocking the achievement of aspirational goals associated with recovery, such as travel. For one participant, since becoming unwell her pets had lost all their beneficial elements.

*Yes the only thing is my future plans revolve around saving up as much money as possible and travelling for as many years as possible which means dogs and cats that I’ve got I won’t be able to keep so *(ID 14, 2 dogs and 2 cats, first circle).

#### Emotional and illness work

When participants talked about the work that their pets did to support them in managing their mental health on a day-to-day basis, narratives about illness and emotional work were conflated at times. Unsurprisingly, pets were rarely implicated in everyday practical work (such as house work) but were considered important in relation to illness-related practical work and emotional work.

Given the consistency of presence and a close physical proximity, pets constituted an instantaneous source of calming, therapeutic benefit for their owners. Pets were a source of physical contact and comfort and a way for individuals to channel their own emotional energy often not available elsewhere.

*Yes, you get comfort from them, because they lick you and all that, and they knead you with their claws and purr at you and all that, so yes, they’re lovely *(ID 8, 2 cats, first circle).

The network benefits associated with pets could be direct or experienced indirectly via pets owned by other people, but whose benefits were transmitted. Pets owned by others in the network could provide solace and support that some participants could not source themselves within their own network. There was also a sense that animals were imbued with intuition for when their owners were feeling unwell to which they behaved accordingly.

*When I’m feeling really low they are wonderful because they won’t leave my side for two days. I will get up and I will let them out to the toilet and I will feed them but I am straight back in bed and I won’t even get myself any food or water and then they’ll just come straight back up and just stay with me until I’m ready to come out of it. They are used to it I suppose* (ID 14, 2 dogs and 2 cats, first circle).

One element of the intimate relationship with pets was their input as a source of practical illness work, notably in relation to distraction and disruption from negative feelings, emotions and untoward symptoms. This finding indicates a therapeutic role beyond that found previously for other long-term conditions. For example, pets could distract their owners from positive symptoms of schizophrenia such as hearing voices, from suicidal ideation or from a general sense of feeling alone.

*But if I’m here and I’m having…having problems with voices and that, erm, it does help me in the sense, you know, I’m not thinking about the voices, I’m just thinking of when I hear the birds singing* (ID 2, 2 birds, first circle).

Pets often introduced a source of humour into difficult situations and were often the only thing that could lift participants’ spirits.

*She, sort of, does random stuff, like climbs on the bars and… stuff [laugh] and things [which distract me] and it’s quite funny watching her what she does because she’s not like a normal hamster *(ID 3, 1 hamster, second circle).

Given this attributed function of distraction and disruption, pets were particularly beneficial in crisis situations. In comparison with other relationships within their network, pets were considered as an omnipotent and constant presence so people could rely on this source of distraction and unconditional support.

*I mean I could always go out, take him out of his, er, hutch, give him a stroke or something [if I needed to]* (ID 3, 1 hamster, second circle).

#### Biographical work

Pets were reported to be important in relation to biographical work given their assistance in managing the stigma associated with the diagnosis and experience of mental illness and by providing ontological security. They also provided self-validation both through their relationship with their owner but also because of a perception that they mediated how other people viewed them.

Pets were identified as having a role in providing routine for their owners. For some, pets encouraged exercise and for others their pets were the only reason they got out of bed in the morning. Through the rituals of feeding, exercise, grooming and caring for their pet a sense of consistent daily routine became embedded in their lives, which participants felt was vital for their wellbeing.

*And I just try and make sure that I walk him, and that, in the mornings….but sometimes I can’t be bothered to do that, but then I think….I..I think about, you know, that it’s not fair if he doesn’t go *(ID 11, 1 dog, third circle).

Participants reported experiencing high levels of felt and enacted stigma related to their diagnosis - even from friends and family. Pets were relevant to an individual’s construction of self and played a unique role in the reduction and management of stigma. For example, pets were seen to accept people for who they were without judgement or resentment. This form of ‘unconditional love’ was an important element of the human-pet dyad, which became increasingly valuable given the vagaries of living with a mental illness.

*And everybody that finds out that you’ve got a mental health problem they will think you’re, you know, off your head and you’re not* (ID 2, 2 birds, first circle).

Participants described how pets (in comparison to human relationships) understood boundaries and knew intuitively when to leave them alone. There was a perception that pets did not hold past behaviours against them and accepted them for who they were. Friends and family members however, often as a result of past behaviours including suicide attempts, overstepped boundaries and made intrusions into their lives that were often not welcomed by the participants included in this study.

*They [pets] don’t look at the scars on your arms, or they don’t question things, and they don’t question where you’ve been* (ID 12, 1 dog, first circle).

Participants reported feeling negatively experienced pressure from friends and family members. This included a perception that friends and family could ask too much of them and pressurise them to recover when they did not feel able to. Having friends and family members rely on them or to ask them for help could be challenging for participants, especially when they were feeling acutely unwell. Complicated dynamics between people in their network could also be stressful to cope with. Relationships with pets were altogether a more simple affair, and they asked very little of each other.

*Well, you know, apart from being fed, they don’t make many demands* (ID 10, 2 cats, first circle).

Others discussed similarities with their pets in relation to their mental health condition (e.g. budgie also having Post-Traumatic Stress Disorder (PTSD)), which may indicate that this identification could be used by participants as a way of managing their own conditions. At the very least, this identification meant that participants did not feel alone in their experiences. Pets were passive recipients of these characteristics, which were projected freely on to them by individuals in a way that appeared to fulfil a specific need to do so.

*I love budgies and every budgie I’ve had I’ve always managed to get it into a position where it will sit on my shoulder and at the moment I’m just training this one because I’m sure he’s got PTSD from living with [friend’s] nan because she used to just chuck things at him in the cage so that’s why the home said we had to get rid of the bird because she wasn’t leaving the room and consequently we took the bird and she’s getting better in the home she’s in. I look after that bird every day, I wake him up, I sit with him and in the evening I’ll sit for a good hour playing with him on his cage or in his cage.* (ID 15, Budgie and goldfish, second circle).

Reciprocity embedded in relationships with pets demarcated such relationships from human ones, which were often not considered reciprocal.

*When he comes and sits up beside you on a night, it’s different, you know, it’s just, like, he needs me as much as I need him, sort of thing.* (ID 9, 1 dog, first circle).

### The hidden work of pets

Successfully caring for a pet could provide a source of validation. Pet owners talked about the pride associated with having a pet that was seen to be well loved and cared for. Given the high levels of unemployment and isolation within the sample, participants had limited other opportunities to develop this form of validation. One participant’s love of animals had led her to the local city farm where she volunteered, which impacted on her confidence. Often, the physical connection with pets was enhanced through mastery such as teaching an animal tricks. Through these relationships with their pets, participants could present themselves to others in a more positive light.

*I mean it’s just a nice feeling to have somebody around that you can l, like, take care of (ID 3, 1 hamster, second circle).*

Despite this perceived value attributed to illness work, pets were unanimously neither incorporated into participants’ discussions with health service providers nor into mental health care planning. Our data indicates that the work undertaken by pets has little salience to those in positions of power in relation to decision making and service provision within health services. Most participants, however, could see the benefit of incorporating pets into these discussions through the development of an holistic understanding of the individual and the production of more relevant and useful care plans.

*Kind of, knowing about your cats and your friends and your family would feed into them knowing you and understanding you a bit better, and…Which would, in turn, feed into how useful the care plan could be. (ID 5, 2 cats, first circle).*

## Discussion

To our knowledge, this is the first qualitative study empirically exploring the role of pets in the social networks of people managing a long-term mental health problem. Using a social network approach incorporating illness work concepts, we identified the attributional meaning attached to pets by those diagnosed with mental health conditions as well as the implicated role of pets in different types of illness work.

Pets contributed, over time, to individuals developing routines that provided emotional and social support. This was set against a backdrop of pets also providing the ability to gain a sense of control inherent to caring for a pet, which was absent in relationships with other network members. This seemed to enable a sense of security and routine to be developed in relationships with pets, which reinforced stable cognitions from the creation of certainty that they could turn to and rely on pets in times of need. With reference to how Giddens [3] used the term, pets provided ontological security through generating a sense of order and continuity to individual experiences and through this close connection provided a sense of meaning to people’s lives. Pets also served as passive recipients of projected characteristics. For example, one participant discussed how her pet also had PTSD, which meant she did not feel alone in her condition and could relate to another network member with whom she perceived to share experiences. In this sense, the work of pets in personal communities provided participants with a seemingly deep and secure relationship, often not available elsewhere within the network or wider community. This became increasingly important given the often uncertain illness trajectory associated with severe mental illness including recovery and periods of crisis.

In terms of the illness work associated with managing mental health, our findings point to the value of pets in illness practical work. This included distraction and disruption from distressing symptoms, such as hearing voices, suicidal thoughts, rumination and facilitating routine and exercise for those who cared for them. Furthermore, pets were implicated in biographical work through their direct impact on managing the stigma associated with mental illness. Pets provided a form of acceptance for their owners and participants considered that by undertaking the tasks associated with being a responsible pet owner, this positively impacted on how others viewed them. These aspects of illness work provide an extension to previous findings about the role of pets for physical illness management [32] and mental health (i.e. a reduction in stress [24] reduced loneliness, [2, 31] and the receipt of social capital [13]). The findings also contrast with previous research that demonstrates the negative impact of pet ownership [38] and of losing a family pet [39].

It is not the intention of this paper to indicate that pets play a more important role for one type of health concern than another, rather that there are nuanced differences in the ways in which people with labels of mental and physical conditions may come to view recovery [40] and the impact that a diagnosis may have on a sense of self [40, 41]. On the face of things, it appears that the participants raised similar themes as those with physical health conditions [32]. However, in relation to the salience of themes with specific regard to mental health, there were clear differences. Participants in this study had more difficult and contentious relationships with others and experienced greater levels of stigma than those included in studies of chronic physical conditions. This increased the perceived importance of their pets, reflecting the added salience of being labelled with a mental health problem as having a greater impact on one’s sense of ‘self’ than physical illnesses, since the surveillance of moral responsibility may be felt more intensely, and levels of isolation and stigma are likely to be greater [40, 41].

### Service implications–the hidden work of pets

The network mapping undertaken as part of this study illuminated the role of pets as a hidden resource for mental health management and supports the idea of a ‘lifestyle’ approach to the management of mental health problems and prevention [42]. The latter involves the incorporation of holistic principles to enhance physical and mental wellbeing, including environmental, behavioural and psychological principles [43] and this study identifies pets as a hidden asset that could be deployed in this regard. However, the value and utility of pets as part of an active point of discussion and resource for people remains invisible within mental health service provision and in the negotiation of individual care plans. A lack of consideration for individual caring responsibilities for pets also represented a source of worry for some of the participants included in this study when they considered the chance of them being in a crisis in the future (e.g. concern for the care of their pet should they become hospitalised). This suggests the need to consider including pets in the care planning process so that service users feel confident that their pets are cared for and returned to them should they not be able to care for them for a period.

Further implications for health services are the inclusion of pets as a topic of discussion, to facilitate healthcare discussions. Previous research suggests that service users feel distanced from healthcare and uninvolved in discussions about services [44, 45]. Taking more creative approaches to care planning discussions, including the use of pets, may be one way of addressing this because of the value, meaning and engagement that individuals have with their companion animals. The study also highlighted the timeliness of incorporating pets into discussions with those in services – particularly about managing mental health over time, with pets considered particularly useful at times of crisis but potentially restrictive when aspirational goals associated with recovery were considered.

### Strengths and limitations

Key strengths of the paper were the utilisation of an established theoretical framework (Corbin and Strauss’s Illness Work) and the comparison with non-pet owning participants. Adopting a qualitative, social network approach provided rich data with which the theoretical ‘illness work’ framework [31] was used to allow participants to describe the unique and distinct role of pets within their personal communities compared with other network members. The authors considered that theme saturation was achieved with the data collected, and participants were sampled to ensure a variety of attitudes were encapsulated into the study. Participants were recruited from within two locations in the UK, included only those cared for withing the community and did not recruit any participants from Black, Asian and minority ethinc communities. It therefore may not be possible to fully transfer findings in terms of typicality to other ethnic groups or other service populations.

## Conclusions

Drawing on an approach incorporating notions of illness work and a personal workforce of support, this study contributes to the understanding of the role of pets in the management of mental health. This was achieved through the identification of the unique role and value of pets in relationships and work associated with managing mental health over time. The implications of this study propose that pets should be considered a main, rather than a marginal source of support, in the management of long-term mental health problems and could be considered as extending more traditional Collaborative Care Models for managing mental health [46]. These insights provide the mental health community with possible areas to target intervention and potential ways in which to better involve service users in service provision through the discussion of valued experiences.

## Appendix 1

Prompt questions for interviews: List of the prompt questions utilised during the interviews with participants.

### Prompt questions for interviews

1. How do your people/pets help you manage your condition day to day?
2. What do people/pets do to help you cope with your illness?
3. Where or to whom do you go to find out more about your illness?
4. Is there anything else that you find useful to help you cope with your illness?
5. When you need advice about, or help with, your diet, who do you go to?
6. When you need advice about, or help with, exercise, who do you go to?
7. Where would you go, or who would you go to, for advice or help with relieving stress?
8. When you need advice about, or help with, medications, who would you turn to?
9. a) Living with a long-term condition often means that you need to do things more slowly, take on additional tasks and other people may need to make compromises that are good for your health. Who in your diagram does these things?
  b) Please describe in detail what you would do on a typical day starting from getting up in the morning.
  Please include tasks and activities that are not related to managing your condition, such as cooking, cleaning, making repairs, etc. Can you tell us how different people/pets on your diagram are involved with different activities?
10. Who do you turn to when you are worried about your illness?
11. a) Looking at your diagram, who do you think you would like to be more involved in helping you with your illness than they are at present?
  b) What and who helps or hinders your care (related to diet/exercise/medication)? Can you think of examples?
12. Who or what (e.g. people/pets) in your diagram gives you emotional support and encouragement? Can you think of examples?
13. Who in your diagram would step in/stand up for you when you do not feel well enough to stand up for yourself?
14. Who among the people or pets in your diagram do you help? How?

## Appendix 2

Information for each participant’s social network including the number of pets, total network size and position of pet within the network.

**Table 4:** Information on study participants

ID Number	Number of pets	Network Size	Position of pet
1	1–Cat	16	First Circle
2	1–birds (2 but combined in network)	6	First circle
3	1–hamster	8	Second circle
4	1–dog	7	Third circle
5	2–cats (2 but combined in network)	7	First circle
6	1–guinea pig	4	First circle
7	1–cat	15	Second circle
8	1–cats (2 but combined in network)	6	First circle
9	1–dog	8	First circle
10	1–cats (2 but combined in network)	10	First circle
11	1–dog	6	Third circle
12	1–dog	10	First circle
13	4–Dog, Parrot, Rabbit, guinea Pig	7	First circle
14	4–Dogs (2), Cats (2)	11	First circle
15	2–Budgies, goldfish	19	Second circle
16	1–Dog	16	First circle
17	1 unknown	19	Not placed on map
18	1 Dog	21	First circle
19	1 Dog	16	First circle
20	1 Cat	21	Second circle
21	10 Birds	11	First circle
22	1 Cat	22	Second circle
23	4–1 rabbit, 2 finches, 1 Hamster	12	Third circle
24	1 (not specified)	22	Not placed on map
25	1 cat	18	First circle

## Abbreviations

AAT: Animal Assisted Therapy
CLAHRC: Collaboration for Leadership in Applied Health Research and Care
CPN: Community Psychiatric Nurse
COREQ: Consolidated Criteria for Reporting Qualitative Research
EQUIP: Enhancing the Quality of User Involved Care Planning in Mental Health Services
NIHR: National Institute for Health Research
UK: United Kingdom
PTSD: Post-Traumatic Stress Disorder
